# Retinal and choroidal changes following corneal collagen cross-linking in keratoconus: a systematic review and meta-analysis of OCT and OCTA studies

**DOI:** 10.1186/s40942-025-00726-w

**Published:** 2025-08-26

**Authors:** Kia Bayat, Aryan Seraj, Parisa Pooyan, Sepehr Feizi, Mozhgan Rezaei Kanavi, Marco A. Zarbin, Hamid Ahmadieh

**Affiliations:** 1 Ophthalmic Research Center, Research Institute for Ophthalmology and Vision Science, Shahid Beheshti University of Medical Sciences, Tehran, Iran; 2 Ocular Tissue Engineering Research Center, Research Institute for Ophthalmology and Vision Science, Shahid Beheshti University of Medical Sciences, Tehran, Iran; 3 School of Medicine, Shahid Beheshti University of Medical Sciences, Tehran, Iran; 4 Institute of Ophthalmology and Visual Science, Rutgers New Jersey Medical School, Newark, NJ USA

**Keywords:** Corneal cross-linking, Keratoconus, Optical coherence tomography, Optical coherence tomography angiography, Retina, Choroid

## Abstract

Corneal collagen cross-linking (CXL) is widely used to halt the progression of keratoconus by biomechanically strengthening the corneal stroma; however, its potential effects on retina and choroid remain unclear. This systematic review and meta-analysis synthesized current evidence on structural and microvascular changes in the posterior segment following CXL in patients with keratoconus, assessed by optical coherence tomography (OCT) and OCT angiography (OCTA). A comprehensive search of PubMed, EMBASE, and Web of Science was performed up to May 24, 2025. Random-effects meta-analysis using Hedges’ g was applied to pool quantitative data. In addition, studies that met the eligibility criteria but lacked sufficient data for quantitative synthesis were qualitatively assessed and included in the descriptive analysis. Ten studies involving 233 eyes from 215 keratoconus patients were included. Meta-analysis demonstrated no significant change in central macular thickness at 1 month (Hedges’s g = -0.15; 95% confidence interval [CI]: -0.44 to 0.13; *p* = 0.30) or 6 months (Hedges’s g = -0.12; 95% CI: -0.47 to 0.22; *p* = 0.48). Subfoveal choroidal thickness also remained unchanged at 1 month (Hedges’s g = -0.14; 95% CI: -0.45 to 0.17; *p* = 0.37). Sensitivity analyses confirmed the robustness of these results. In the qualitative synthesis, parameters demonstrated overall stability, aside from a few exceptions. In conclusion, current evidence suggests that CXL does not result in clinically meaningful changes in posterior segment structure or microvasculature in keratoconus patients. These findings support the posterior segment safety of CXL.

**Supplementary Information:**

The online version contains supplementary material available at 10.1186/s40942-025-00726-w.

## Key messages

**What is known:**

• Corneal collagen crosslinking (CXL) is the established treatment to halt progression of keratoconus by inducing stromal collagen crosslinks through UVA irradiation and riboflavin application.

**What is new:**

• CXL has minimal lasting impact on retinal and choroidal structures, likely due to UV-absorbing ocular media and riboflavin’s photoprotective properties.

• Reported transient changes (e.g., parafoveal and perifoveal thinning, inner retinal layer thickening, sectoral alterations in the deep capillary plexus, reduced total avascular complex, subfoveal choroidal thickening, and decreased choroidal vascularity index) resolve without lasting sequelae and are more likely attributable to postoperative inflammation or medication effects than to phototoxicity.

• Differences in some retinal outcomes (e.g., macular RNFL and GCL thickness) between conventional and accelerated CXL may reflect protocol-specific responses.

**Supplementary Information:**

The online version contains supplementary material available at 10.1186/s40942-025-00726-w.

## Introduction

Keratoconus is a progressive ectatic disorder characterized by biomechanical weakening of the corneal stromal collagen matrix [1, 2]. Histopathological studies reveal disrupted collagen lamellae, reduced collagen content, and diminished natural cross-linking within keratoconic corneas, culminating in decreased tensile strength and structural integrity [1, 3, 4].

Corneal collagen cross-linking (CXL) has emerged as a minimally invasive intervention designed to halt keratoconus progression by enhancing the biomechanical stability of the corneal stroma [5]. The conventional Dresden protocol involves epithelial debridement followed by saturation of the cornea with riboflavin, which functions as a photosensitizer [6]. Subsequent exposure to ultraviolet-A (UV-A) light (315–400 nm wavelength) induces photochemical reactions that promote the formation of new covalent cross-links between collagen fibrils [7]. This process enhances corneal rigidity and tensile strength, thereby stabilizing the cornea and arresting ectatic progression [8]. Robust clinical evidence supports CXL as the standard of care for progressive keratoconus, demonstrating its efficacy in flattening steep corneal curvature and reducing the necessity for corneal transplantation [9].

Advances in optical imaging have revolutionized the visualization of posterior segment anatomy and vasculature with unprecedented resolution. Optical coherence tomography (OCT) is a noninvasive imaging modality employing low-coherence interferometry to produce high-resolution, depth-resolved cross-sectional images of the retina and choroid [10, 11]. More recently, OCT angiography (OCTA) has enabled the noninvasive assessment of retinal and choroidal microvasculature by registering sequential OCT scans to generate en face angiograms of superficial and deep retinal plexuses as well as the choriocapillaris [12, 13].

While CXL is primarily used for the cornea, some studies have explored the potential adverse effects of UV-A in other areas, including the retina and choroid. Recent OCT and OCTA studies examining adult keratoconus patients post-CXL have reported heterogeneous results, with some demonstrating significant changes [14], while others observe minimal or no impact [15].

Despite these observations, the clinical relevance and reproducibility of posterior segment alterations following CXL for keratoconus remain uncertain. Variability in imaging techniques and follow-up duration complicate interpretation, and no consensus has been established regarding CXL-induced retinal or choroidal modifications. This ambiguity highlights the critical need for a systematic synthesis of existing evidence.

In this study, we aim to provide a comprehensive review of retinal and choroidal changes in patients with a history of CXL treatment for keratoconus, thereby clarifying the potential impact of this widely adopted intervention on posterior segment integrity.

## Methods

This systematic review was conducted in strict adherence to the Preferred Reporting Items for Systematic Reviews and Meta-Analyses (PRISMA) guidelines [16]. The study protocol was prospectively registered with the International Prospective Register of Systematic Reviews (PROSPERO) under the registration number CRD420251071880.

### Search strategy

A comprehensive literature search was performed on May 24, 2025 to identify relevant studies investigating structural and microvascular alterations in the posterior segment of the keratoconus eye following CXL, detected via OCT or OCTA. The databases queried included Web of Science, EMBASE, and PubMed/MEDLINE. The search employed a combination of controlled vocabulary and keywords, including “keratoconus,” “corneal ectasia,” “corneal cross-linking,” “optical coherence tomography,” and “optical coherence tomography angiography.” Detailed search strategies tailored to each database are provided in Supplementary File 1.

To enhance the comprehensiveness of the search and minimize the omission of eligible publications, forward and backward citation tracking of included studies was carried out. No limitations were applied regarding publication year or geographical origin. Eligible study designs included cross-sectional, case-control, and cohort studies. Excluded from the review were experimental studies, editorials, correspondence, conference proceedings, review articles, and case series or case reports.

### Study screening and selection

All retrieved citations were imported into EndNote 20 for reference management. Automated and manual deduplication processes were employed by two independent reviewers (K.B. and P.P.). The same reviewers then independently screened titles and abstracts to exclude clearly irrelevant records. Full-text articles were subsequently assessed for eligibility. Any disagreements were resolved through discussion and consensus.

### Inclusion and exclusion criteria

Studies were eligible for inclusion if they met the following criteria: (1) original research articles published in English; (2) enrollment of adult patients with a documented history of CXL for keratoconus; (3) absence of concurrent ocular pathologies or history of ocular surgery rather than CXL; and (4) the use of OCT and/or OCTA to evaluate retina and choroid after CXL. No exclusion criteria were applied based on disease severity.

**Fig. 1:**
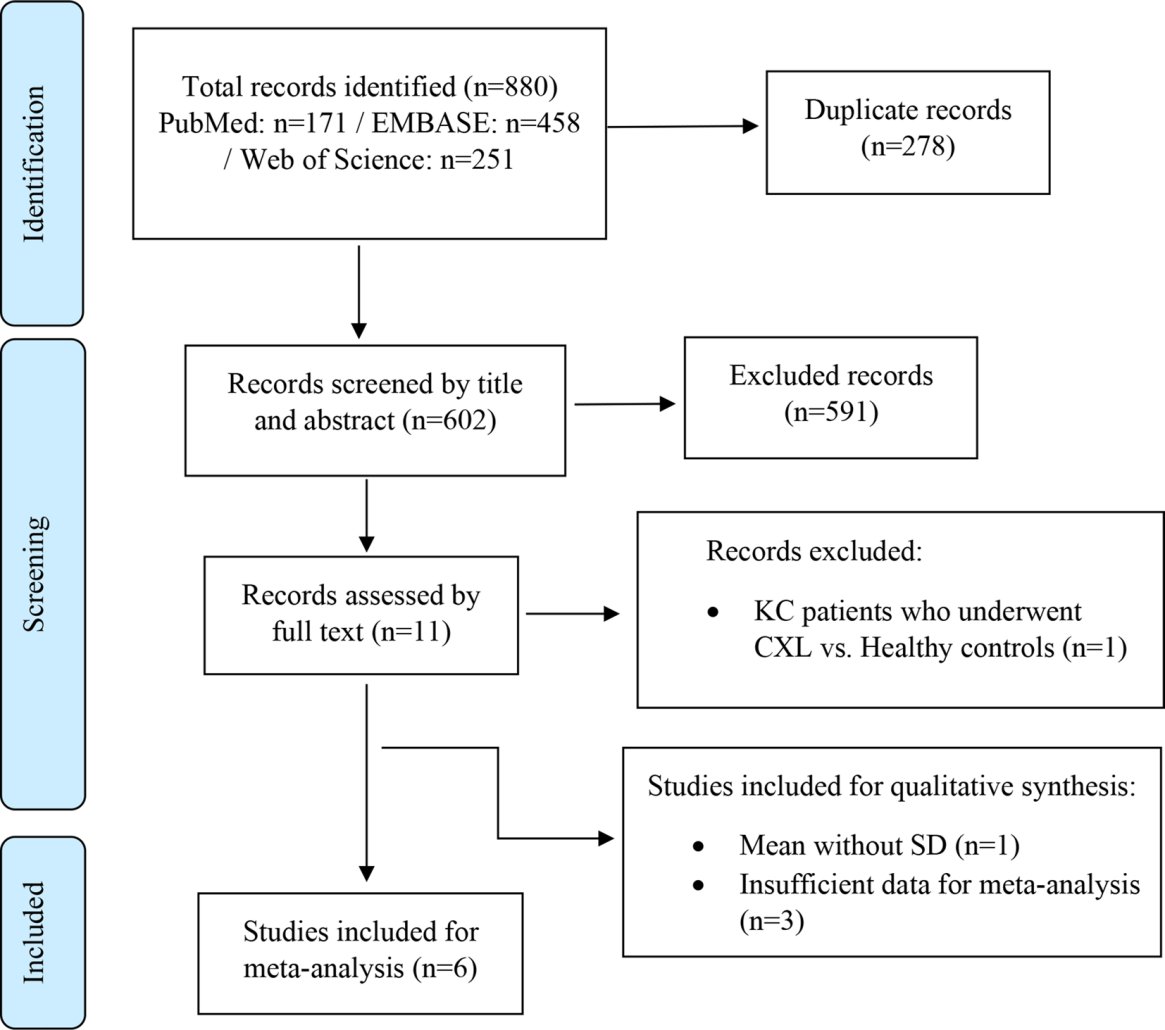
PRISMA flowchart

One study [17] was excluded because it only compared postCXL patients to healthy controls, without evaluating pre versus postoperative outcomes. Other reasons for study exclusion from the meta-analysis included the lack of reported standard deviation (SD) values in one study [18] and insufficient data for quantitative analysis in three studies [19–21]. All discrepancies in the inclusion process were resolved through consensus. The study selection process is visually presented in the PRISMA flow diagram (Fig. 1).

### Data extraction

Two reviewers (K.B. and A.S.) independently designed and implemented a standardized data extraction form. Extracted data included: (1) study characteristics: year of publication, study design, first author, and country of origin; (2) participant demographics: total sample size, age, and sex distribution; (3) baseline ophthalmologic examinations: best corrected visual acuity (BCVA), refraction, keratometry readings, and thinnest corneal thickness; (4) keratoconus severity; (5) CXL protocol employed; (6) imaging device specifications: model and manufacturer of OCT or OCTA instruments; and (7) quantitative imaging parameters: macular structure, macular microvasculature, peripapillary and optic nerve, and choroidal parameters. All differences in extracted data were reconciled through discussion until agreement was reached.

### Risk of bias assessment

The methodological quality of the included studies was independently assessed by two reviewers (K.B. and P.P.) using the Newcastle-Ottawa Scale (NOS) [22], which evaluates selection, comparability, and outcome (for cohort studies) or exposure (for case-control studies). The scale assigns a maximum of nine stars, and studies achieving a score of seven or more were considered to have low risk of bias. Any discrepancies in scoring were resolved through consensus. Full results of the risk of bias assessment are presented in Supplementary File 2.

### Statistical analysis

Meta-analytic computations were performed using a random-effects model with Hedges’ g as the effect size metric. Restricted maximum likelihood (REML) estimation was employed to account for between-study heterogeneity. Statistical heterogeneity was quantified using the tau-squared (τ²) and I-squared (I²) statistics, and statistical significance was assessed with Cochran’s Q test (*p* < 0.05). A Galbraith plot was constructed to visually evaluate the heterogeneity.

Sensitivity analyses were conducted by sequentially excluding individual studies to assess the robustness of findings. Publication bias was examined through Begg’s rank correlation test and Egger’s regression test, and further explored visually through funnel plot asymmetry. All statistical analyses were performed using Stata software (version 17), with a p-value of less than 0.05 considered statistically significant.

## Results

### Study selection

A total of 880 records were identified through an extensive database search, comprising 251 from Web of Science, 458 from EMBASE, and 171 records from PubMed. After duplicate entries were eliminated, 602 unique articles remained for screening. Based on the evaluation of titles and abstracts, 11 articles were deemed potentially eligible and underwent full-text review. Ultimately, 6 studies, encompassing 132 eyes from 119 participants, satisfied the inclusion criteria and were incorporated into the quantitative synthesis for meta-analysis. An additional 4 studies, while not meeting all criteria for quantitative inclusion, contained relevant data and were therefore included in the qualitative synthesis. Altogether, this systematic review analyzed 10 original studies, covering a total of 233 eyes from 215 patients. The detailed selection process is depicted in Fig. 1.

### Study characteristics

This systematic review included 10 cross-sectional studies [14, 15, 18–21, 23–26]. In terms of geographic distribution, four studies were conducted in Iran [15, 18, 19, 26], three in Turkey [14, 21, 24], and Israel [25], Germany [20], and Brazil [23] contributed one study each. Eye selection methodology varied; seven studies assessed one eye per participant [15, 19–21, 23, 25, 26], while three studies included mixed eye selection approaches [14, 18, 24]. The included studies reported mean participant ages ranging from 20.1 to 32.0 years, with female representation spanning 23.5–56.0%. Baseline spherical equivalent was available from two studies, with mean values of -2.83 D and − 3.61 D [20, 21]. Preoperative average keratometry values varied from 46.2 to 52.0 D, while thinnest corneal thickness measurements ranged from 451.2 to 480.4 μm. Keratoconus severity stratification was reported in only two studies, both demonstrating predominantly mild-to-moderate disease: one cohort comprised 92.8% stage 1 or 2 cases [24], while another reported 90% stage 2 involvement [18]. Eight studies followed the conventional Dresden protocol using epithelium-off techniques with 3 mW/cm² irradiation for 30 min. Two studies employed alternative approaches: one utilized a non-Dresden conventional epithelium-off method [25], while the other implemented an accelerated protocol with 9-minute irradiation [21]. All included studies performed epithelial removal, and no epithelium-on techniques were documented. Various models of OCT devices were employed across studies, including Heidelberg [19, 21, 23, 24], Optovue [15, 18, 20, 26], Topcon [14], and Zeiss [25]. The included studies evaluated a wide range of OCT-derived structural and vascular parameters such as macular structural measurements in forms of overall retinal thickness [19], central macular thickness (CMT) [14, 15, 20, 21, 23, 25, 26], parafoveal [14, 20, 26] and perifoveal thickness [20, 26], inner and outer retinal layer thickness [21, 26], as well as individual macular layers including macular retinal nerve fiber layer (RNFL) [14, 21], ganglion cell layer (GCL) [14, 21], inner plexiform layer (IPL) [21], inner nuclear layer (INL) [21], outer plexiform layer (OPL) [21], outer nuclear layer (ONL) [21], and retinal pigment epithelium (RPE) [21]; macular microvascular measurements in terms of vessel density (VD) of the superficial (SCP) and deep (DCP) capillary plexuses [14, 18, 19], avascular complex [19], and foveal avascular zone (FAZ) metrics [14]; peripapillary and optic nerve parameters including peripapillary RNFL thickness [19] and optic nerve head (ONH) vessel density [19]; choroidal parameters in terms of subfoveal choroidal thickness (SFCT) [14, 15, 24], choroidal thickness at 500 μm and 1500 μm from the foveal center [24], parafoveal choroidal thickness [14], choroidal volume [15], choroidal vascularity index (CVI) [24], and vessel density in the choriocapillaris (CC) [14] (Table 1).

**Table 1:** Study characteristics

Study	Country	OCT Device	Patients / Eyes (No.)	Female percentage (%)	Age (Years)	Preop BCVA (logMAR)	Preop thinnest corneal thickness (µm)	Preop average K (D)
**Quantitative**								
Ayaz, 2022	Turkey	Topcon	20/27	—	—	0.37 ± 0.16	—	—
Nasrollahi, 2021	Iran	Optovue	25/25	56.0	28.72 ± 6.77	0.06 ± 0.05	—	—
Doganay, 2024	Turkey	Heidelberg	22/28	—	23.2 ± 4.1	—	459 ± 42.5	46.3 ± 4.01
Mirzaei, 2018	Iran	Optovue	21/21	42.9	20.9 ± 5.95	—	—	—
Barbisan, 2018	Brazil	Heidelberg	17/17	35.3	22.17 ± 5.0	0.47 ± 0.12	—	—
Goldich, 2010	Israel	Zeiss	14/14	42.9	28.2 ± 5.9	0.21 ± 0.1	—	46.2 ± 2.8
**Qualitative**								
Taheri, 2025	Iran	Optovue	25/30	52.0	24.0	—	451.2	46.57
Bamdad, 2024	Iran	Heidelberg	22/22	40.9	20.09 ± 6.11	—	480.36 ± 35.72	—
Lazaridis, 2020	Germany	Optovue	17/17	23.5	32 ± 8	0.26 ± 0.24	—	—
Ozsaygili, 2021	Turkey	Heidelberg	32/32	34.4	23.9 ± 5.4	0.23 ± 0.15	455.46 ± 35.95	52.04 ± 4.37

### Data synthesis

#### Meta-analysis

The meta-analysis of pooled data demonstrated no statistically significant differences between preoperative CMT measurements and those measured at 1-month and 6-month follow-up visits. Specifically, for the 1-month postoperative exam, the pooled effect size was Hedges’s g = -0.15 (95% confidence interval [CI]: -0.44 to 0.13; *p* = 0.30). Similarly, for the 6-month postoperative evaluation, the effect size was Hedges’s g = -0.12 (95% CI: -0.47 to 0.22; *p* = 0.48). No heterogeneity was observed for either time point, as indicated by I² values of 0.00% (*p* = 0.86 for 1 month; *p* = 0.63 for 6 months).

Similarly, analysis of pooled SFCT data comparing baseline and 1-month postoperative measurements demonstrated no significant changes. The calculated effect size was Hedges’s g = -0.14 (95% CI: -0.45 to 0.17; *p* = 0.37), with no evidence of heterogeneity (I² = 0.00%, *p* = 0.98).

**Fig. 2:**
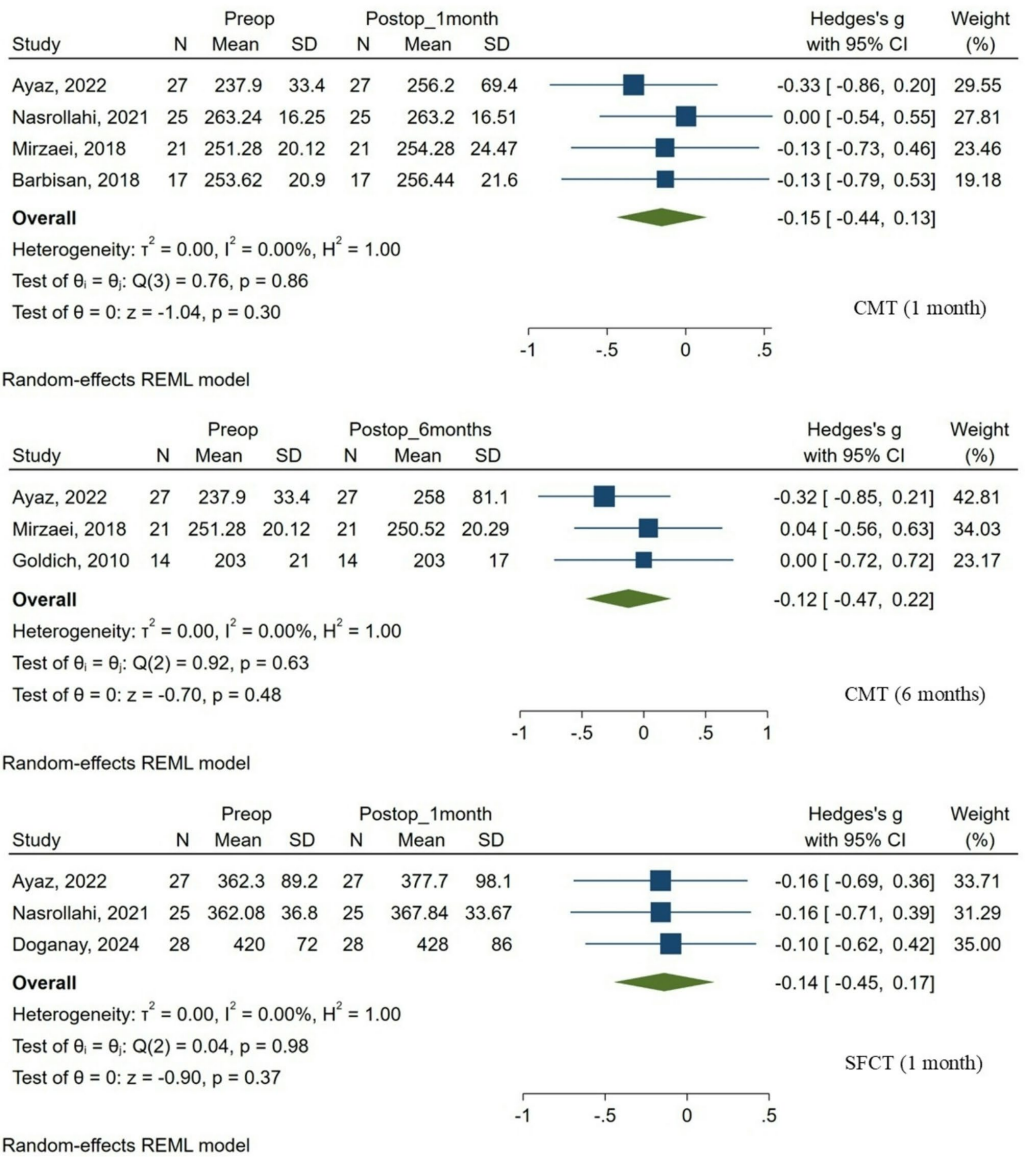
Forest plots depicting retinal and choroidal changes following CXL in patients with keratoconus

The detailed outcomes of these meta-analyses are presented in Table 2 and illustrated in Fig. 2.

**Table 2:** Meta-analysis summary of retinal and choroidal changes following CXL in patients with keratoconus

OCT parameter	No. of studies	Effect sizes (Diff: Postop – Preop)		Heterogeneity		
		Hedges’s g (95% CI)	*p*-value	x^2^	I^2^, %	*p*-value
CMT 1 month	4	-0.15 (-0.44, 0.13)	0.30	0.76	0.00	0.86
CMT 6 months	3	-0.12 (-0.47, 0.22)	0.48	0.92	0.00	0.63
SFCT 1 month	3	-0.14 (-0.45, 0.17)	0.37	0.04	0.00	0.98

**Fig. 3:**
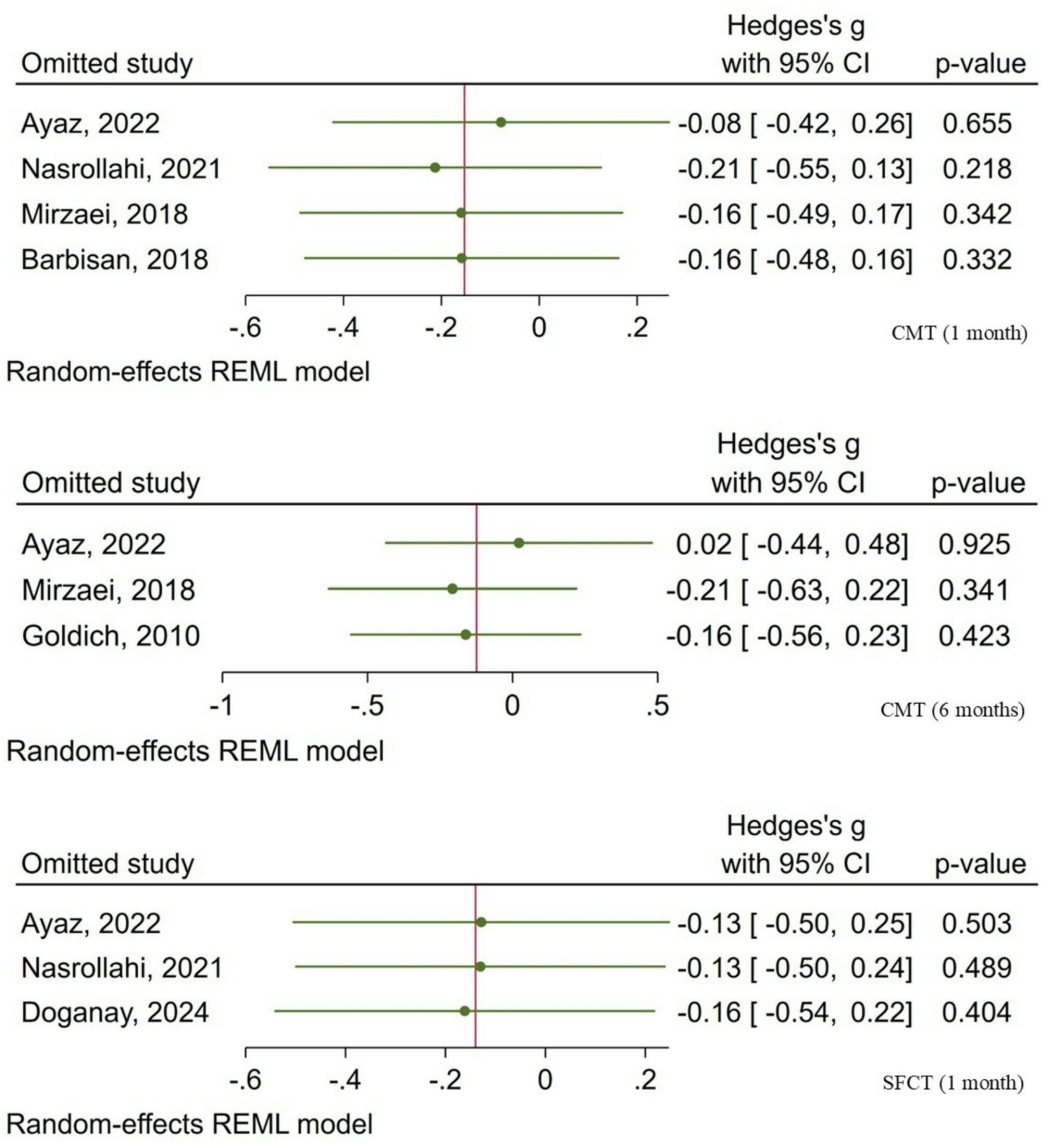
Sensitivity analyses of retinal and choroidal changes following CXL in patients with keratoconus

Sensitivity analyses were conducted to evaluate the stability of the meta-analytic estimates. Sequential exclusion of individual studies did not produce any substantial alterations in the pooled effect sizes, confirming the robustness of the findings (Fig. 3).

**Fig. 4:**
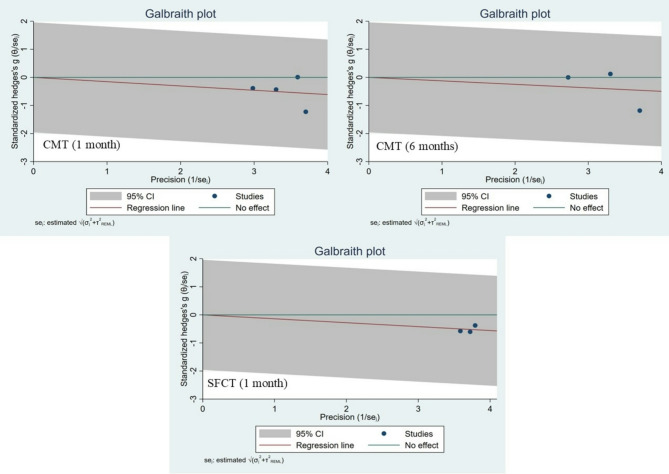
Galbraith plots of retinal and choroidal changes following CXL in patients with keratoconus

Galbraith plots were generated to further assess heterogeneity across the included studies (Fig. 4). The analyses confirmed the absence of significant heterogeneity among the examined parameters.

**Fig. 5:**
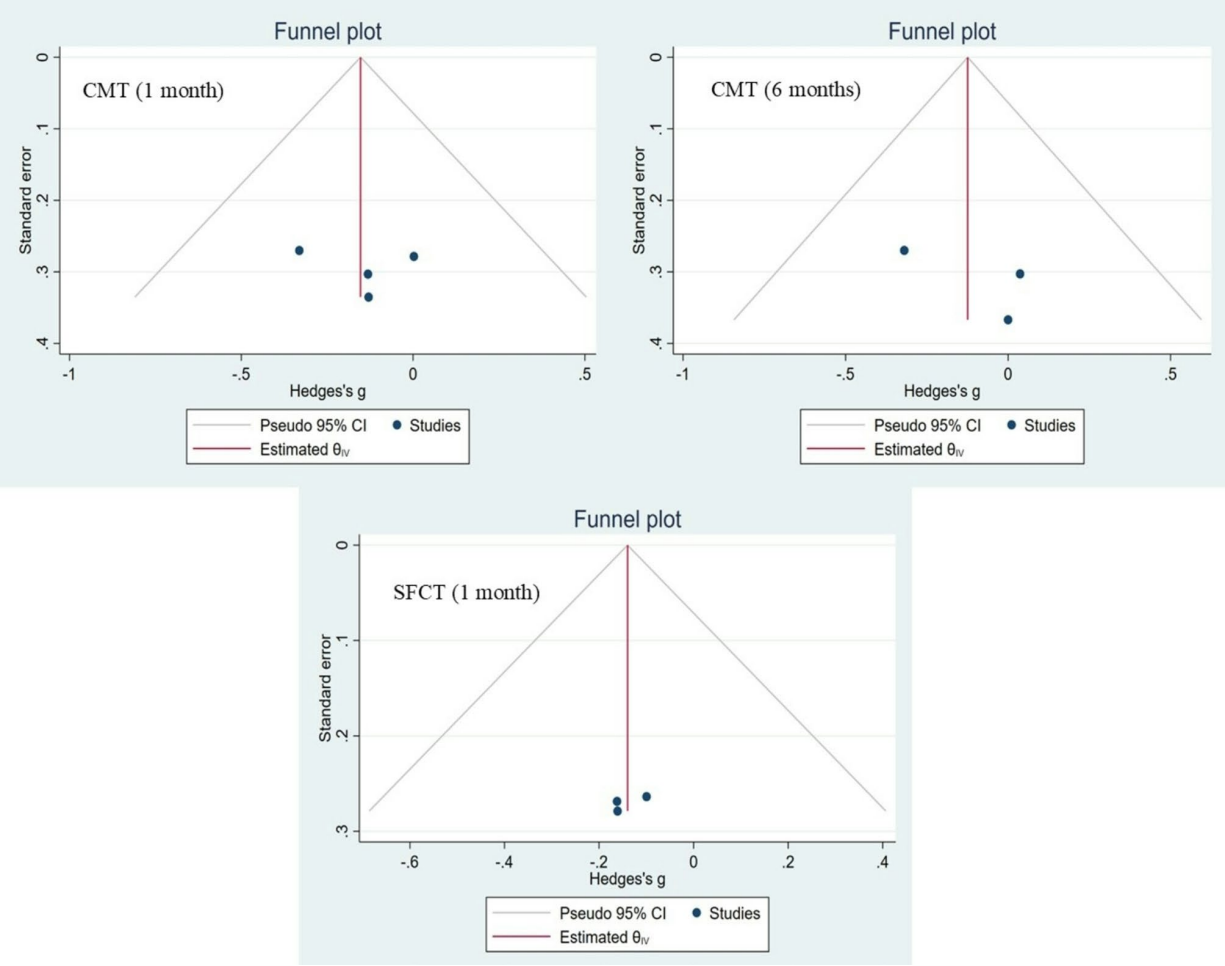
Funnel plots of retinal and choroidal changes following CXL in patients with keratoconus

Potential publication bias was assessed using multiple complementary approaches. Funnel plots were visually inspected for asymmetry (Fig. 5), and formal statistical tests, including Egger’s regression and Begg’s rank correlation, were performed (Supplementary File 3). Both visual and statistical evaluations revealed no indication of publication bias across the analyses.

### Qualitative synthesis

#### Macula

Macular structural parameters demonstrated general stability across all time points (Table 3).

**Table 3:** Comprehensive overview of macular structural alterations following CXL in patients with keratoconus

Parameter	3 days	1 week	1 month	3 months	6 months	12 months
Average retinal thickness (Total, as well as superior and inferior quadrants)	—	—	Bamdad: ↔	Bamdad: ↔	—	—
CMT	—	Barbisan, Ayaz: ↔ Lazaridis (2 weeks): ↔	Barbisan, Nasrollahi, Ayaz, Mirzaei: ↔ Lazaridis (6 weeks): ↔	—	Goldich, Ayaz, Mirzaei: ↔	Ozsaygili, Goldich: ↔
Parafoveal thickness	—	Ayaz: ↔ Lazaridis (2weeks): ↓	Ayaz, Mirzaei: ↔ Lazaridis (6 weeks): ↓	—	Ayaz, Mirzaei: ↔	—
Perifoveal thickness	—	Lazaridis (2weeks): ↓	Mirzaei: ↔ Lazaridis (6 weeks): ↓	—	Mirzaei: ↔	—
Outer-retina thickness	—	—	Mirzaei: fovea, parafovea, perifovea: ↔	—	Mirzaei: fovea, parafovea, perifovea: ↔	Ozsaygili: ↔
Inner-retina thickness	—	—	Mirzaei: fovea, parafovea, perifovea: ↑	—	Mirzaei: fovea, parafovea, perifovea: ↔	Ozsaygili: ↔
Macular RNFL thickness	—	Ayaz: center: ↑, parafovea: ↔	Ayaz: center: ↑, parafovea: ↔	—	Ayaz: center: ↑, parafovea: ↔	Ozsaygili: ↔
GCL thickness	—	Ayaz: center, parafovea: ↔	Ayaz: center, parafovea: ↔	—	Ayaz: center: ↔, parafovea: ↓	Ozsaygili: ↔
Other macular segments ^a^	—	—	—	—	—	Ozsaygili: ↔

^a^ Inner plexiform layer, Inner nuclear layer, Outer plexiform layer, Outer nuclear layer, Retinal pigment epithelium

Note: Up arrows (↑) indicate significant increase; down arrows (↓) demonstrate significant reduction; and bidirectional arrows (↔) exhibit no significant change

Bamdad et al. [19] reported no change in average retinal thickness (total, superior, inferior quadrants) at 1 and 3 months post-CXL.

CMT remained unchanged at 1 week [14, 23], 2 weeks [20], 4 weeks [14, 15, 23, 26], 6 weeks [20], 6 months [14, 25, 26], and 12 months [21, 25] postoperatively.

Parafoveal thickness assessments showed no significant changes at multiple time points (1 week, 1 month, and 6 months) [14, 26]. However, Lazaridis [20] documented a reduction in parafoveal thickness at both 2 and 6 weeks following the procedure. Perifoveal thickness remained stable at 1-month and 6-months follow-up in the study by Mirzaei et al. [26], whereas Lazaridis et al. [20] reported thinning at 2 and 6 weeks postoperatively.

Outer retinal thickness measurements across foveal, parafoveal, and perifoveal regions remained unchanged throughout the follow-up period [21, 26]. In contrast, inner retinal thickness increased at 1 month in all regions [26], followed by stabilization at subsequent visits [21, 26].

Macular RNFL thickness showed regional variability. Ayaz et al. [14] reported a significant increase in central RNFL thickness at 1 week, 1 month, and 6 months postoperatively, while parafoveal RNFL thickness remained unchanged during these intervals. However, Ozsaygili et al. [21], who employed an accelerated CXL protocol, observed no significant changes in macular RNFL at 12 months.

Regarding the GCL, Ayaz et al. [14] consistently reported stable measurements in both the central and parafoveal regions at 1 week and 1 month and a reduction in parafoveal GCL thickness at 6 months. On the contrary, Ozsaygili et al. [21] documented no significant changes in GCL at 12 months.

Other macular segments, including the IPL, INL, OPL, ONL, and RPE, exhibited no postoperative alterations in thickness according to the study by Ozsaygili et al. [21]

Analysis of macular microvasculature demonstrated general preservation of VD in both the SCP and DCP throughout the follow-up periods (Table 4).

**Table 4:** Comprehensive overview of macular microvascular alterations following CXL in patients with keratoconus

Parameter	3 days	1 week	1 month	3 months	6 months	12 months
SCP VD	—	Ayaz: center, parafovea: ↔	Ayaz: center, parafovea: ↔ Taheri: Whole image, superior-hemi, inferior-hemi, fovea, parafovea, and perifovea: ↔ Bamdad: total: ↔, large: ↔, capillary: ↔	Bamdad: total: ↔, large: ↓, capillary: ↑	Ayaz: center, parafovea: ↔ Taheri: whole image, inferior-hemi, fovea, parafovea, and perifovea: ↔, superior-hemi: ↓	—
DCP VD	—	Ayaz: center: ↑ parafovea: ↔	Ayaz: center: ↑ parafovea: ↔ Taheri: whole image, superior-hemi, inferior-hemi, parafovea, and perifovea: ↔, fovea: ↓ Bamdad: total: ↔, large: ↓, capillary: ↔	Bamdad: total: ↔, large: ↓, capillary: ↑	Ayaz: center: ↑ parafovea: ↔ Taheri: whole image, superior-hemi, inferior-hemi, parafovea, and perifovea: ↔, fovea: ↓	—
Avascular complex (Total, large, and capillary)	—	—	Bamdad: ↔	Bamdad: ↔	—	—
FAZ VD (SCP and DCP)	—	Ayaz: ↔	Ayaz: ↔	—	Ayaz: ↔	—

Note: Up arrows (↑) indicate significant increase; down arrows (↓) demonstrate significant reduction; and bidirectional arrows (↔) exhibit no significant change

In the SCP, Ayaz et al. [14] reported stable VD in both the central and parafoveal regions at 1 week, 1 month, and 6 months post-CXL. Taheri et al. [18] similarly documented no significant changes in whole image, superior-hemi, inferior-hemi, foveal, parafoveal, and perifoveal regions at 1 and 3 months, except for a significant reduction in the superior-hemi sector at 6 months. Bamdad et al. [19] observed stability in total, large, and capillary vessel components at 1 month, with a reduction in large vessels and an increase in capillary density by 3 months.

Two studies reported different findings regarding changes in DCP of the foveal region. Ayaz et al. [14] observed an increase in VD in the central region at 1 week, 1 month, and 6 months, with no significant changes in the parafoveal areas. In contrast, Taheri et al. [18] reported largely stable DCP measurements across most sectors but noted a localized reduction in foveal VD at both 1 month and 6 months. Additionally, Bamdad et al. [19] observed no change in total VD and a decrease in large VD at both 1 and 3 months, while capillary VD remained stable at 1 month and increased at 3 months.

The avascular complex, including total, large, and capillary components, remained unchanged at 1 and 3 months postoperatively [19]. Similarly, no significant alterations were observed in FAZ vessel density in either the SCP or DCP at 1 week, 1 month, or 6 months following CXL [14].

### Peripapillary area

RNFL thickness (global and quadrant-specific measurements) were stable at 1 month and 3 months postoperatively [19]. In addition, ONH VD assessments revealed no alterations in the SCP, DCP, or avascular complex at 1 month [19]. By 3 months, however, the total avascular complex density demonstrated a significant decrease while other parameters remained unchanged (Table 5).

**Table 5:** Comprehensive overview of peripapillary area alterations following CXL in patients with keratoconus

Parameter	3 days	1 week	1 month	3 months	6 months	12 months
Peripapillary RNFL (Global & all quadrants)	—	—	Bamdad: ↔	Bamdad: ↔	—	—
ONH VD (SCP, DCP, and avascular complex (total, large, and capillary))	—	—	Bamdad: ↔	Bamdad: total avascular complex: ↓ Other measurements: ↔	—	—

Note: Down arrows (↓) demonstrate significant reduction; and bidirectional arrows (↔) exhibit no significant change

### Choroid

Choroidal analysis demonstrated variable findings across different parameters (Table 6).

**Table 6:** Comprehensive overview of choroidal alterations following CXL in patients with keratoconus

Parameter	3 days	1 week	1 month	3 months	6 months	12 months
SFCT	Doganay: ↔	Ayaz: ↔	Ayaz, Nasrollahi, Doganay: ↔	Doganay: ↑	Ayaz: ↔	—
CT at 500 μm (quadrant-specific)	Doganay: ↔	—	Doganay: ↔	Doganay: ↔	—	—
CT at 1500 μm (quadrant-specific)	Doganay: ↔	—	Doganay: ↔	Doganay: nasal, inferior, superior: ↔, temporal: ↓	—	—
Parafoveal CT	—	Ayaz: ↔	Ayaz: ↔	—	Ayaz: ↔	—
Choroidal volume	—	—	Nasrollahi: ↔	—	—	—
CVI	Doganay: Horizontal: ↓ Vertical: ↔	—	Doganay: ↔	Doganay: ↔	—	—
CC VD (Center & parafovea)	—	Ayaz: ↔	Ayaz: ↔	—	Ayaz: ↔	—

Note: Up arrows (↑) indicate significant increase; down arrows (↓) demonstrate significant reduction; and bidirectional arrows (↔) exhibit no significant change

SFCT remained stable at 3 days [24], 1 week [14], 1 month [14, 15, 24], and 6 months [14]. An increase in SFCT was observed at 3 months in the study by Doganay et al. [24]

Quadrant-specific choroidal thickness at 500 μm showed no significant changes at any evaluated time point [24]. Similarly, quadrant-specific measurements at 1500 μm remained stable at 3 days and 1 month. However, a decrease was observed in the temporal quadrant at 3 months despite the nasal, inferior, and superior quadrants remained unchanged [24].

Parafoveal choroidal thickness remained unchanged at 1 week, 1 month, and 6 months in the study by Ayaz et al. [14] Choroidal volume demonstrated no significant change at 1 month [15].

In the study by Doganay et al. [24], the CVI showed a decrease in the horizontal sector at 3 days, while the vertical sector remained unchanged. CVI remained stable at subsequent follow-up assessments at 1 and 3 months.

CC VD at both the center and parafovea was stable at 1 week, 1 month, and 6 months [14].

## Discussion

The accumulated evidence indicates that corneal CXL has minimal effects on the posterior segment of the eye. While a few studies have documented transient macular thinning in the parafoveal and perifoveal regions, these alterations were short-lived. Inner retinal layers increased after CXL, which may be due to a reactive response of neurons and glial cells within the inner retinal layers in response to phototoxicity [27–29]. One study using conventional CXL reported increased central macular RNFL thickness from 1 week through 6 months postoperatively, accompanied by reduced parafoveal GCL thickness at the 6-month timepoint [14]. Conversely, a study employing accelerated CXL found no significant alterations in either macular RNFL or GCL thickness at 12-month follow-up [21]. Although differing follow-up durations may account for the observed disparities, these findings may alternatively suggest distinct retinal responses between conventional and accelerated protocols, highlighting the need for future comparative studies. In terms of retinal microvasculature, sectoral changes were observed in the DCP after CXL. The differences observed in certain measurements across studies may be attributed to variations in geographical location, OCT device used, and the age profiles of the study populations. A reduction in the total avascular complex at the ONH was reported in a single study [19]. Furthermore, SFCT thickening and a reduction in CVI were also documented in a separate study [24]. Read et al. [30] demonstrated that a short period of morning light therapy can induce a significant increase in choroidal thickness in healthy adults, supporting the idea that light exposure may influence choroidal vascular responses. Although the spectral properties and purpose of light exposure differ from those used during UV-A-mediated corneal cross-linking, the observed thickening in both settings may share common neurovascular or circadian regulatory mechanisms [30].

Importantly, these anatomical findings are supported by functional assessments. Multifocal electroretinogram (mfERG) responses demonstrated transient reductions at two weeks post-CXL, returning to baseline by six weeks [31]. In addition, visual acuity remained stable following CXL [14, 31].

The general preservation of posterior segment anatomy following CXL is biologically plausible and is likely attributable to the photoprotective properties of the eye. During the CXL procedure, the cornea is saturated with riboflavin and subsequently irradiated with UV-A light [32] Riboflavin serves a critical UV-protective role, with a peak absorption at 370 nm [33]. The standard Dresden protocol employs UV-A at this wavelength (370 nm, 3 mW/cm²), aligning with riboflavin’s absorption spectrum [34]. Consequently, in eyes with corneal thicknesses ≥ 400 μm, the majority of UV-A energy is absorbed in the anterior stroma [35, 36]. Only a minimal fraction of UV-A reaches the corneal endothelium, and even less penetrates deeper ocular tissues, including the retina and choroid [19, 37, 38]. Spoerl et al. [38] calculated that in a 400 μm–thick, riboflavin-saturated cornea, the UV-A irradiance at the endothelial level is approximately 0.18 mW/cm², less than half the known damage threshold for endothelial cells. As the lens and vitreous further attenuate UV-A transmission, retinal irradiance remains several orders of magnitude below phototoxic levels [38].

Beyond photoprotection, corneal biomechanics contribute to the safety profile of CXL. Cross-linking predominantly affects the anterior 300 μm of the stroma, resulting in localized stiffening and flattening of the cornea [38]. This biomechanical effect halts the progression of ectasia but does not alter axial globe length or induce structural changes in the posterior segment. Any corneal curvature changes following CXL are typically minor (< 2 diopters) and unlikely to significantly affect ocular geometry at the retinal level [15].

Nonetheless, the focal posterior segment changes observed in a subset of eyes may be explained by indirect mechanisms such as (1) inflammation: CXL, especially epithelium-off protocols, provokes a transient but robust anterior inflammatory cascade. Epithelial debridement and UVA/riboflavin treatment induce keratocyte apoptosis and release pro-inflammatory mediators (IL-1β, IL-6, TNF-α, etc.) in the corneal stroma and tear film [39–41]. Laser flare photometry shows a slight but sustained post-CXL breakdown of the blood–aqueous barrier for weeks to months [39]. In theory these cytokines and any residual UVA-generated free radicals could diffuse posteriorly or activate intraocular immune cells. Indeed, transient light-sensitivity syndrome after CXL has been attributed to keratocyte-derived cytokines causing ciliary-body inflammation [42–50]; (2) medications: postoperative regimens commonly include topical antibiotics and corticosteroids. Steroid-induced elevations in intraocular pressure may transiently alter choroidal circulation or retinal autoregulation [51–53]. However, given the brief and tapering course of corticosteroids following CXL, any associated changes in macular or choroidal thickness are likely minimal and reversible.

### Limitations and future directions

Several limitations should be considered when interpreting the findings of this systematic review and meta-analysis. First, many of the included studies had small sample sizes, which limits the statistical power to detect subtle or infrequent changes. Second, heterogeneity existed among studies with respect to imaging protocols, device models, and segmentation algorithms, which can potentially affect the consistency of measurements. Third, the follow-up duration was limited in most studies, with none extending beyond 12 months; thus, the long-term effects of CXL on the posterior segment remain unclear.

Future research should focus on investigating the effects of different CXL protocols, specifically distinguishing between conventional and accelerated techniques. Each of these protocols may have distinct impacts on posterior segment structures, warranting separate and in-depth examination. Studies with larger cohorts, standardized imaging methodologies, and extended follow-up are essential to further elucidate these effects.

## Conclusion

This comprehensive review supports the conclusion that CXL is a procedure whose impact primarily involves the anterior segment, with negligible impact on posterior segment anatomy. Natural UV absorption by the cornea, lens, and vitreous, the UV-protective role of riboflavin, and minimal changes in corneal biomechanics following CXL, collectively provide a clear explanation for why direct injury to the posterior segment is unlikely. Observed alterations appear to be transient and may reflect downstream effects of postoperative inflammation or medication use. However, as the sample sizes in the included studies were limited, these findings should be interpreted with appropriate caution and continued monitoring in larger, long-term studies is warranted to fully exclude the possibility of delayed effects.

## Supplementary Information

Below is the link to the electronic supplementary material.

Supplementary Material 1

Supplementary Material 2

Supplementary Material 3

## Data Availability

No datasets were generated or analysed during the current study.

## Declarations

### Generative AI and AI-assisted technologies in the writing process

After completing the writing and editing of the manuscript, the authors used ChatGPT exclusively for the final revision and minor adjustments. Following the use of this tool, the authors reviewed and edited the content as needed and take full responsibility for the content of the published article.
