# Supplementary material for: Retinal and choroidal changes following corneal collagen cross-linking in keratoconus: a systematic review and meta-analysis of OCT and OCTA studies
Source: Int J Retina Vitreous. 2025 Aug 26;11:97. doi: 10.1186/s40942-025-00726-w (PMC12379409; doi:10.1186/s40942-025-00726-w)
Supplement: Supplementary file 1 — Supplementary Material 1 [file 40942_2025_726_MOESM1_ESM.docx]

Duplicates: 278

PubMed: 171 articles

#1: (((Keratoconus[MeSH Terms]) OR (keratoconus[Title/Abstract])) OR ("corneal ectasia"[Title/Abstract])) OR ("corneal ectatic disorders"[Title/Abstract])

#2: (((Tomography, Optical Coherence[MeSH Terms]) OR ("optical coherence tomography angiography"[Title/Abstract]))) OR ("optical coherence tomography"[Title/Abstract])

#3: (((Corneal Cross-Linking[MeSH Terms]) OR ("Corneal Cross-Linking"[Title/Abstract])) OR ("Corneal Collagen Cross-Linking"[Title/Abstract])) OR ("Cross-Linking"[Title/Abstract])

#1 AND #2 AND #3

EMBASE: 458 articles

#1: 'keratoconus'/exp OR keratoconus:ab,ti OR 'corneal ectasia':ab,ti OR 'corneal ectatic disorders':ab,ti

#2: 'optical coherence tomography angiography'/exp OR 'optical coherence tomography angiography device'/exp OR 'optical coherence tomography angiography':ab,ti OR 'optical coherence tomography'/exp OR 'optical coherence tomography device'/exp OR 'optical coherence tomography':ab,ti

#3: 'corneal crosslinking'/exp OR 'corneal collagen cross-linking device'/exp OR 'corneal crosslinking':ab,ti OR 'corneal collagen cross-linking':ab,ti OR 'cross linking'/exp OR 'cross linking':ab,ti

#1 AND #2 AND #3

Web of Science: 251 articles

#1: ((TS=(keratoconus)) OR TS=("corneal ectasia")) OR TS=("corneal ectatic disorders")

#2: (TS=("optical coherence tomography angiography")) OR TS=("optical coherence tomography")

#3: ((TS=("Corneal Cross-Linking")) OR TS=("Corneal Collagen Cross-Linking")) OR TS=("Cross-Linking")

#1 AND #2 AND #3
