# Supplementary material for: Retinal and choroidal changes following corneal collagen cross-linking in keratoconus: a systematic review and meta-analysis of OCT and OCTA studies
Source: Int J Retina Vitreous. 2025 Aug 26;11:97. doi: 10.1186/s40942-025-00726-w (PMC12379409; doi:10.1186/s40942-025-00726-w)
Supplement: Supplementary file 2 — Supplementary Material 2 [file 40942_2025_726_MOESM2_ESM.docx]

S2. Risk of bias assessment of the cross-sectional studies with Newcastle-Ottawa scale (NOS)

| **Study** | **Selection (Maximum 5 stars)** | | | | **Comparability (Maximum 1 stars)** | **Outcome (Maximum 3 stars)** | | **Total score (out of 9)** |
| --- | --- | --- | --- | --- | --- | --- | --- | --- |
|  | **Representativeness of the cases** | **Sample size** | **Non-Response rate** | **Ascertainment of the screening/surveillance tool** | **The potential confounders were investigated by subgroup analysis or multivariable analysis** | **Assessment of the outcome** | **Statistical test** |  |
| Nasrollahi, 2021 | * |  | * | ** | * | ** | * | 8 |
| Doganay, 2024 | * |  | * | ** | * | ** |  | 7 |
| Ayaz, 2022 | * |  | * | ** | * | ** |  | 7 |
| Mirzaei, 2018 | * |  | * | ** | * | ** |  | 7 |
| Barbisan, 2018 | * |  | * | ** | * | ** |  | 7 |
| Goldich, 2010 | * |  | * | ** | * | ** |  | 7 |
| Taheri, 2025 | * |  | * | ** | * | ** | * | 8 |
| Bamdad, 2024 | * |  | * | ** | * | ** | * | 8 |
| Lazaridis, 2020 | * |  | * | ** | * | ** | * | 8 |
| Ozsaygili, 2021 | * |  | * | ** | * | ** |  | 7 |
