# Supplementary material for: Retinal and choroidal changes following corneal collagen cross-linking in keratoconus: a systematic review and meta-analysis of OCT and OCTA studies
Source: Int J Retina Vitreous. 2025 Aug 26;11:97. doi: 10.1186/s40942-025-00726-w (PMC12379409; doi:10.1186/s40942-025-00726-w)
Supplement: Supplementary file 3 — Supplementary Material 3 [file 40942_2025_726_MOESM3_ESM.docx]

S3. Publication bias analysis

| **Parameter** | **Regression Test** | | **Rank Correlation Test** | |
| --- | --- | --- | --- | --- |
|  | **T** | **p-value** | **Kendall's tau** | **p-value** |
| CMT (1 month) | 1.40 | 0.818 | 0.00 | 1.000 |
| CMT (6 months) | 3.25 | 0.490 | 1.00 | 1.000 |
| SFCT (1 month) | -3.51 | 0.888 | -1.00 | 1.000 |
